# Role of Sonification and Rhythmic Auditory Cueing for Enhancing Gait Associated Deficits Induced by Neurotoxic Cancer Therapies: A Perspective on Auditory Neuroprosthetics

**DOI:** 10.3389/fneur.2019.00021

**Published:** 2019-01-29

**Authors:** Shashank Ghai, Ishan Ghai

**Affiliations:** ^1^Institute of Sports Science, Leibniz University Hannover, Hanover, Germany; ^2^Consultation Division, Program Management Discovery Sciences, RSGBIOGEN, New Delhi, India

**Keywords:** cueing, chemotherapy, stability, rehabilitation, performance, balance, perception

## Abstract

Patients undergoing chemotherapy, radiotherapy, and immunotherapy experience neurotoxic changes in the central and peripheral nervous system. These neurotoxic changes adversely affect functioning in the sensory, motor, and cognitive domains. Thereby, considerably affecting autonomic activities like gait and posture. Recent evidence from a range of systematic reviews and meta-analyses have suggested the beneficial influence of music-based external auditory stimulations i.e., rhythmic auditory cueing and real-time auditory feedback (sonification) on gait and postural stability in population groups will balance disorders. This perspective explores the conjunct implications of auditory stimulations during cancer treatment to simultaneously reduce gait and posture related deficits. Underlying neurophysiological mechanisms by which auditory stimulations might influence motor performance have been discussed. Prompt recognition of this sensorimotor training strategy in future studies can have a widespread impact on patient care in all areas of oncology.

## Introduction

Pharmacological treatment of cancer is varying dramatically with benefits for better patient outcomes and ease, but also with new toxicity profiles (1–3). Neurotoxicity is an unavoidable complication of life-saving cancer treatments, such as chemotherapy, radiotherapy, and immunotherapy (4, 5). Typically, treatment with immunotherapeutic agents involves activation of the body's own immune system for targeting malignant cells (6) (Table 1). During the treatment cross-adverse reactions with existing neural cells result in heightened neurotoxicity (7–9). Topp et al. (10) for instance, reported that approximately >50% of patients receiving Blinatumomab for acute B-lymphoblastic leukemia exhibited movement disorders, encephalopathic changes, cerebellar dysfunctions, and seizures. Similarly, chemotherapy acts by instigating damage to the structural composition of the DNA, and by also disrupting DNA repair and microtubule functioning. During its functioning the chemotherapeutic agents impart non-specific damage on the cells of the nervous system, thereby resulting in neurotoxicity (9) (Table 1). The most commonly used class of chemotherapy drugs include Vinca alkaloids. This class of drugs has been reported to disrupt microtubule functioning, promote degeneration and axonal atrophy in dosages more than 2 mg/m^3^ (11). Furthermore, radiotherapy inhibits cell division and promotes neurotoxicity by inducing vascular damage, hormonal disruption, alteration in cytokine expression, neural stem cell deletion, neural fibrosis (12, 13) (Table 1) [for a detailed review see (14)]. Several factors can influence the extent of neurotoxicity induced by radiation therapy i.e., volume of brain irradiated, fraction (>200cGy), cumulative radiation dosage (<5,000cGy), simultaneous administration of chemotherapy, administration of therapy in age groups <7 years old or more than 60 years old and pre-existence of stroke (15). Despite precarious planning to irradiate specific parts and minimize neuropathy, radiation-induced neurotoxicity is still prevalent in several parts of the neural axis (12).

**Table 1:** Pharmacological interventions for cancer treatment and associated neurotoxic effects.

**Treatments**	**Drugs**	**Neurotoxic effects**
Immunotherapy	Bispecific antibodies (Blinatumomab), Monoclonal antibodies (Trastuzumab, Brentuximab, Rituximab, Ramucirumab, Bevacizumab), Cellular treatments (Chimeric antigen receptor-T cells), Checkpoint inhibitors (Nivolumab Pembrolizumab, Ipilimumab), Tyrosine kinase inhibitors (Imatinib, Dasatinib, Ponatinib, Erlotinib, Pazopanib, Aflibercept, Idelalisib, Sorafenib, Sunitinib), Interferon alfa, Recombinant Interleukin 2	Peripheral nervous system: Gullian Barre syndrome, Myasthenia gravis, sensorimotor peripheral neuropathy, multifocal plexopathy/neuropathy, autonomic neuropathy, phrenic nerve palsy, cranial nerve palsy (optic, hypoglossal, facial nerve) Central nervous system: Aseptic meningitis, encephalitis, transverse myelitis, neurosarcoidosis, posterior reversible leukoencephalopathy syndrome, Vogt Harada Koyanagi syndrome, neurosarcoidosis, demyelination, vasculitis encephalopathy, generalized seizures, convulsions
Chemotherapy	Taxanes (Paclitaxel, Docetaxel), Epothilones (Ixabepilone), Platinum derived compounds (Cisplatin, Carboplatin, Oxaliplatin), Immunomodulatory drugs (Lenalidomide, Bortezomib, Thalidomide), Inhibitor of topoisomerase (Etoposide), Vinka alkaloids (Vincristine, Vindesine, Vinblastine, Vinorelbine), Metalloids (Arsenic), Alkylating agents (Procarbazine, Ifosfamide), Antimetabolites (5-Fluorouracil, Capecitabine, Gemcitabine, Fludarabine, Cytarabine), Farnesyltransferase inhibitors (Tipifarnib), Antiprotozoal and anthelmintic (Suramin)	Peripheral nervous system: Lhermitte's sign, (painful) sensory peripheral neuropathy, muscle cramps, post infusion parenthesias, sensorimotor peripheral neuropathy, mononeuroptherapy, cranial nerve palsy, autonomic neuropathy, myalgia, proximal motor weakness, lumbosacral radiculopathy, painful axonal peripheral neuropathy, ataxia, orthostatic hypotension, intrinsic hand muscle weakness, brachial plexopathy Central nervous system: Encephalopathy, headache, stroke, seizures, cortical blindness, ataxia, athetosis, parkinsonism, radiculomyeloencephalopathy, cerebellar dysfunctions, leukoencephalopathy, inflammatory leukoencephalopathy, stupor, somnolence, aseptic meningitis, myelopathy, ocular toxicity, blurred vision
Radiotherapy	-	Peripheral nervous system: Lumbosacral plexopathy and polyradiculopathy, brachial plexopathy, Lhermitte's sign, radiation myelopathy, dysthesia, motor neuron syndrome, muscle atrophy, fasciculations, areflexia Central nervous system: Encephalopathy, Bulbar palsy, cranial nerve injury, optic neuropathy, cochlear damage, radiation-induced central nervous system tumors (glioma, meningioma, vestibular schwannoma), diffused cerebral injury, stenosis/occlusion of extracranial or intracranial cerebral arteries, stroke-like migraine attack after radiation therapy (SMART syndrome), radiation necrosis

There are several pathophysiological mechanisms by which neurotoxicity can be induced. For instance, therapeutic interventions can impart direct damage to the neuron, glia, and modify the cerebral microvasculature (8, 16–18). Moreover, pathological analysis has also suggested that onset of neural necrosis, axonal degeneration due to microtubular and secondary myelin disruptions (19), can result in central and peripheral nervous system neurotoxicity. Although, several sensory, motor, and cognitive deficits have been discussed in the published literature that can result due to neurotoxicity. In this present perspective our objectives are:

a) Outline the impact of cancer treatment-induced neurotoxicity on gait and posture.

b) Discuss the applicability of music-based external auditory stimulations for facilitating gait and postural recovery in cancer patients.

## Motor Deficits (Gait and Posture)

Research has conclusively demonstrated that joint dysfunctions in sensory, motor and cognitive domains due to neurotoxicity can affect activities of daily living, such as gait (5, 20, 21), posture (22), and promote falls. Epidemiological evidence suggests that the majority of the diagnosed patients are geriatrics i.e., 60–70 years old (23, 24). Spoelstra et al. (25), for instance, reported that geriatric patients with a history of cancer were more likely to fall (33%) as compared to patients with no history of cancer (29%). This higher risk of fall can be due to joint additional neurological deficits imposed by drug-induced neurotoxicity and an age-associated neurological decline (2, 25). Studies analyzing the spatiotemporal gait parameters have also reported larger decrements in gait performance for cancer patients (2, 20, 26). Marshall et al. (2), reported a significantly reduced gait velocity, step length, and an increased duration in timed up and go test in patients with cancer as compared to their healthy counterparts (5, 27). Similarly, kinematic discrepancies during gait performance are also documented. Wright et al. (28) analyzed gait performance (3-D motion analysis, EMG) following treatment for acute lymphoblastic leukemia. The authors reported a significant reduction in peak hip extension, knee flexion during the loading phase, plantarflexion during pre-swing, dorsiflexion during initial heel contact, lower ankle moments, and power outputs. The authors also reported that the patients exhibited excessive co-activations and an atypical “out of phase” motor unit firing of gastrocnemius during the late swing and premature firing of tibialis anterior during terminal stance.

Monfort et al. (22) too in a longitudinal analysis reported a significant decrease in balance (center of pressure perturbations in medioateral direction) in breast cancer patients receiving taxane-based chemotherapy. The authors further correlated this decrease in balance with patient-reported outcomes i.e., EORTC QLQ-CIPN20 subscales (European Organization for Research and treatment of Cancer Quality of Life Questionnaire Chemotherapy Induced Peripheral Neuropathy) i.e., increased pain, fatigue, and disruption in physical functioning reported with the treatment progression.

## Cognitive Deficits

In addition to the motor deficits, patients receiving cancer treatment also exhibit heightened cognitive deficits [see chemobrain or chemofog (29)]. These deficits can persist years after the treatment and can considerably affect a patient's quality of life (30). A wide range of cognitive disorders are manifested by patients i.e., disruptions in executive functions, multitasking, concentration, attentional allocation, even memory recall, visuospatial function, and more (29–31). The pathophysiological changes which account for such deficits include white matter abnormality, regional brain volume differences in superior and middle frontal gyri, parahippocampal gyrus, cingulate gyrus, and precuneus (32, 33). Silverman et al. (34), in a PET study, reported that breast cancer patients who received chemotherapy 5–10 years prior had differences in inferior frontal gyrus, contralateral posterior cerebellum, and left inferior frontal gyrus. The authors also implied the onset of cognitive overload by reporting a larger activation pattern of frontal cortical structures i.e., pre-frontal cortex during a memory task (34). This decline in cognitive performance due to adverse neurotoxic effects of oncologic therapy in our opinion might be amplified when coupled with an age-associated decline in cognition. This, then, might promote a major decline in cognitive performance, further affecting autonomic functions such as posture, gait (35). For instance, this reduced cognitive functioning might limit a patient's ability to effectively allocate attentional resources for instance in high-stress environments and instigate falls (36, 37).

## Sensory Deficits

A wide range of sensory deficits are accounted in patients due to neurotoxic effects on the nervous system (8). Evidence of optic neuropathy have been extensively documented due to radiotherapy, intra-arterial administration of drugs such as Carmustine, Oxaliplatin, Tamoxifen, and more (38, 39). Likewise, deficits in vestibular (40), and proprioceptive signaling (41), are also well reported. Vincent et al. (41) for instance, reported that administration of Oxaliplatin drug promoted the onset of movement disorders. The authors suggested that possibly neurotoxic changes impaired specific ionic current channels (NaPIC) on the sensory terminals of muscle proprioceptors further leading to a modified sensory encoding which could have affected motor functioning (41). Additionally, axonal degeneration of sensory neurons, which promotes receptor denervation, have also been associated with sensorimotor aberrations that affect motor execution (41–43). Bibi (44), for instance, reported that cancer therapy-induced neurotoxic changes can also promote pervasive deterioration in the autonomic mechanisms for sensory gating and sensory memory mechanisms. This contextual decline in the available state of sensory information might affect the state of a system to integrate sensorimotor information and develop internal models (45–47). Here, a mismatch incongruency of sensorimotor information or a decrease in the quality of perceptual information could promote sensorimotor deficits, further affecting motor planning, execution during gait, and postural performance (48, 49).

## Conventional Rehabilitation Interventions

A few rehabilitation strategies have been discussed in the published literature that can enhance gait and balance dysfunctions in patients with cancer. These strategies include physiotherapy, physical exercises, virtual reality and more (21, 50, 51) (see Table 2). Moreover, to the best of our knowledge, only one recent systematic review has analyzed the influence of exercise rehabilitation interventions for managing deficits in gait and postural stability in cancer patients undergoing chemotherapy (21). Despite having a high prevalence for inducing fall-related morbidity and mortality (63), such a limited amount of research is a matter of concern for medical practitioners dealing with cancer patients. Therefore, the development of additional rehabilitation interventions that can be applied as an adjunct to conventional pharmacological interventions is strongly warranted.

**Table 2:** Conventional rehabilitation approaches for managing gait and postural deficits associated with neurotoxicity.

**Disorders**	**Interventions**
Gait	Physiotherapy (52)
	Physical exercise (53, 54)
	Virtual reality (obstacle crossing) (51)
	Sensorimotor balance training (55)
	Transcutaneous electrical stimulation (56)
	Joint stabilizers (56, 57)
Postural stability (static and dynamic)	Physiotherapy (58)
	Aerobic endurance training (55)
	Strength training (resistive TheraBand) (55, 59)
	Impact training (60)
	Home-based exercise programs (61)
	Virtual reality (obstacle crossing) (51)
	Closed kinematic chain exercises (62)
	Core stability ball exercises (62)
	Dynamic balance training (ankle point to point reach task) (51)
	Sensorimotor balance training (55)
	Transcutaneous electrical stimulation (56)
	Joint stabilizers (56, 57)

## Prospective Role of Music-Based Therapies: External Auditory Stimulations

Music therapy has been extensively studied in cancer management [for detailed reviews see (64–66)]. This therapy has been reported to decrease pain, stress, anxiety associated with cancer treatment and has also been documented to improve mood, relaxation, and quality of life (66). The studies predominantly deal with either active or passive types of music therapies (64–66). Here, the active therapy signifies playing musical instruments, improvisation, singing, and passive therapy signify listening to music, imagination (2, 3). Although the outcomes of these cumulative studies comprehend the beneficial psychological aspects of music therapy, the aim of this present study is to explore as to how motor rehabilitation might be facilitated by the application of music-based auditory stimulations?

Several studies have reported that a large component of motor (re)learning is dependent upon the extent of sensorimotor integration (67, 68). Here, amplification of sensorimotor representations by enhancing the salience of sensory afferent information while minimally engaging the deficit cognitive resources should be a major objective (69–71). This enhanced sensorimotor representations of body schematics and executed movements could facilitate the development of efficient internal models (46, 72). Thereby, enhancing the system's ability to acquire, process, and execute a skill in an efficient manner (73–75). In the published literature, movement sonification and rhythmic auditory cueing are two well-studied auditory stimulations that have been demonstrated to incur beneficial effects in motor performance by jointly targeting sensorimotor and cognitive deficits (76–83).

Rhythmic auditory cueing can be defined as a repetitive isosynchronous auditory stimulation applied with an aim to simultaneously synchronize motor execution (74, 84). Real-time kinematic auditory feedback (movement sonification) on the other hand is a comparatively new approach (85). Such type of an intervention involves mapping of movement parameters on the sound components, such as pitch, amplitude with a very minimal or no latency (72) [for differential effects of auditory cueing and sonification please see (82)]. Recent systematic reviews and meta-analyses have conclusively demonstrated the benefits of these auditory stimulations on gait and postural stability with aging (77), in neurological disorders such as stroke (82, 83, 86), parkinsonism (78), cerebral palsy (80), and multiple sclerosis (81). Findings from these reviews can have widespread implications for counteracting neurotoxicity related motor deficits in cancer patients.

For instance, rhythmic auditory cueing has been reported to enhance gait, and postural stability performance across all age groups (77). We have previously stated that the majority of the affected cancer population groups are geriatrics and that this factor according to several studies accounts for the majority of fall-related morbidity and mortality (25). Likewise, stroke, a common neurotoxic manifestation also account for widespread movement, cognitive disorders (2). Ghai (82) has demonstrated that both rhythmic and real-time auditory stimulations can benefit stroke patients in recovering their motor and cognitive performances. Additionally, we also presume that damage induced by white matter deficits, which are a prominent manifestation of neurotoxicity can also be supplemented by the application of auditory stimulations (32, 87). Ghai and Ghai (81) recently demonstrated the beneficial effects of auditory cueing on patients with multiple sclerosis (a multifocal white matter disease). The authors stated evidence which supports the possibility of white matter re-organization with auditory-motor training [see (88)].

**Figure 1:**
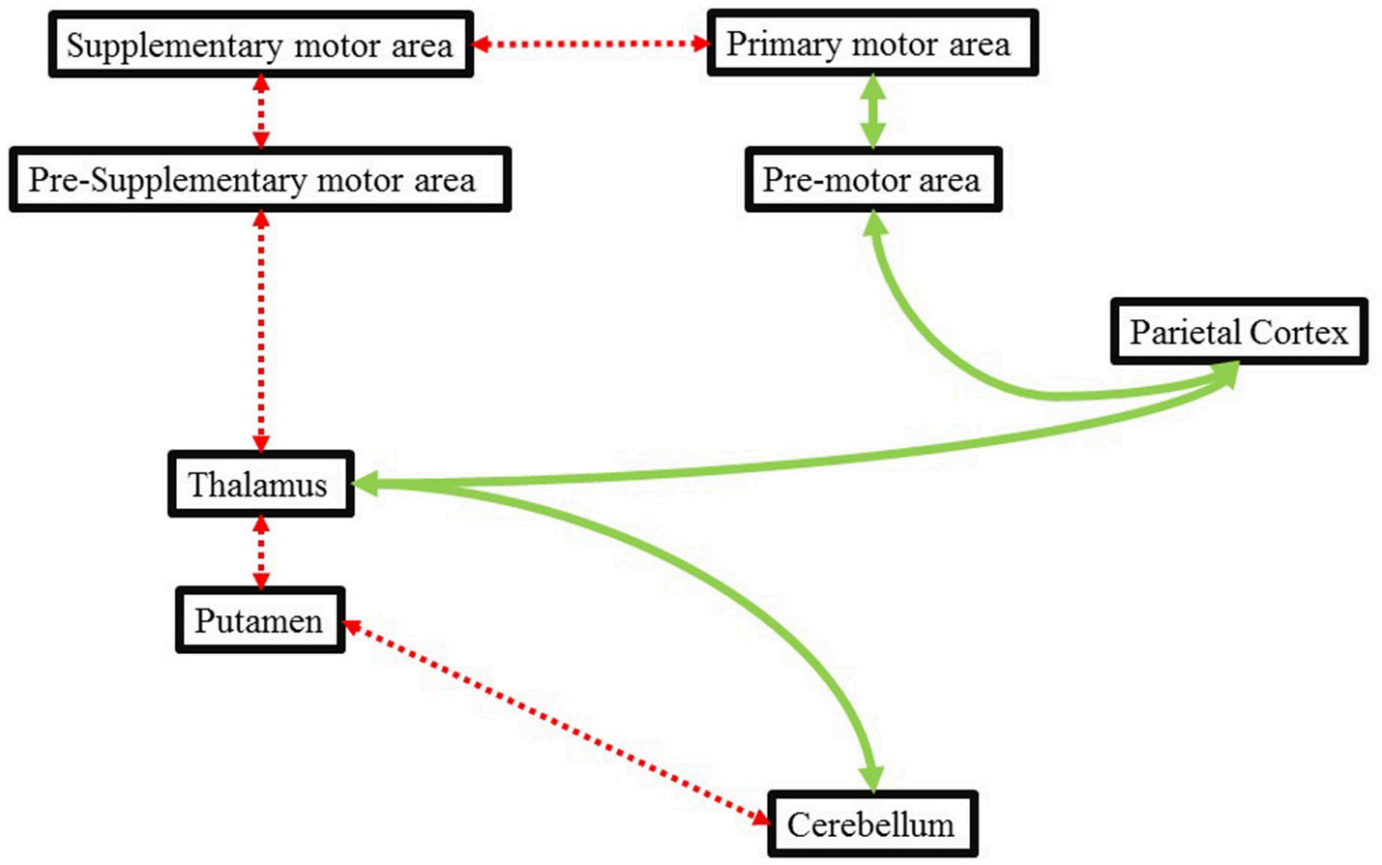
Illustrating the deficit internal cueing pathways (dotted red line) and how external cueing might bypass this deficit pathway via an alternative route (green line). Adapted from Nombela et al. (91) and Ghai et al. (78).

Likewise, Ghai et al. (78), demonstrated the beneficial effects of auditory cueing on movement disorders exhibited during parkinsonism. Chemotherapy, for instance with Metoclopramide (dopamine receptor antagonist) has been associated with inhibition of D_2_ receptors in putamen (89). This disruption has been reported to result in movement disorders which are identical to that exhibited by a patient in Parkinson's disease (90). Here, dysfunctions between the striatopallidal projections could affect the internal timing mechanism of a patient in a similar manner as of a patient with Parkinson's disease. In this instance, the application of external auditory stimulations could assist in movement execution by providing an external cue to time movements. The external cueing can effectively bypass the deficit internal cueing pathway (Cerebellum-putamen-thalamus-pre supplementary motor area-supplementary motor area-primary motor area) through an alternative preserved pathway between (cerebellum-thalamus-parietal cortex-premotor area-primary motor area) and facilitate motor activity (91) (Figure 1).

Furthermore, we presume that the auditory stimulations could counteract sensory-perceptual deficits i.e., hearing, visual loss by enhancing the salience of sensory afferent information and aiding in the development of sensorimotor representations. For instance, Schmitz et al. (92) in a neuroimaging study reported that observation of a convergent sensory feedback can enhance activations in frontoparietal networks, action observation system i.e., superior temporal sulcus, Broadman area 44, 6, insula, precentral gyrus, cerebellum, thalamus, and basal ganglia (92). The activations in these areas are associated with biological motion perception, thereby suggesting an enhancement in sensorimotor representation that might strengthen the perceptual analysis of a movement, ultimately resulting in efficient motor planning and execution (92).

Recent evidence has also demonstrated that auditory stimulations can even facilitate proprioceptive perceptions (93). Ghai et al. (94) demonstrated that concurrent auditory feedback can facilitate enhancements in knee-proprioception. Hasegawa et al. (95) too demonstrated that auditory biofeedback training resulted in enhanced spatiotemporal components of postural stability. Therefore, practical implications can be derived for cancer survivors, where deficits in proprioceptive perceptions are quite prominent (94, 96). According to Hasegawa et al. (95), auditory-motor training promoted a challenging environment that could have facilitated proprioceptive integration [for further insights on neuroimaging data see (97)]. Additional mechanisms by which auditory stimulations can facilitate motor performance are that they can provide explicit guidance to time/execute movements (94), reduce variability in musculoskeletal co-activation (98, 99), provide error feedback (100), enhance auditory-motor imagery (101, 102), allow cortical re-organization (103, 104), facilitate neural plasticity (105, 106), and even facilitate neural regeneration (107–109).

We would also like to draw the reader's attention toward literature suggesting how auditory stimulations might act by counteracting deficits in cognitive processing. Firstly, auditory stimulations have been suggested to strengthen attentional allocation (97). This might allow a patient to effectively switch between different tasks at hand without experiencing cognitive overload and/or movement failure. Secondly, enhanced cross-modal processing between auditory and proprioceptive signals can also circumvent cognitive overload and alleviate motor performance (94, 110). Thirdly, adjoining auditory stimulations with music can be an additional way to overcome cognitive deficits. For instance, coupling the auditory stimulations with musical mnemonics might facilitate synchronization of the oscillatory network in the prefrontal regions (111). Here, Thaut et al. (111) has reported that mnemonics might facilitate “deep encoding” during the acquisition phase of learning and might also amplify the internal timings of neural dynamics in the brain which are normally degraded by demyelination process in multiple sclerosis [also see (81)]. As demyelination is also a prominent neurotoxic manifestation of radiotherapy (8), transferrable beneficial effects on cognitive performance could be expected. Moreover, recent research also suggests that in addition to reducing cognitive overload in patients with stroke, the external auditory cueing via music might facilitate, reorganize deficit cortical structures (107–109). For instance, merging the external auditory stimuli with music can allow facilitation of neural network including prefrontal, and limbic cortex this, in turn, has been associated with cognitive and emotional recovery (109). Likewise, incorporating the component of music with external auditory stimulations might yield additional benefits in terms of reducing anxiety and stress (112). Studies have demonstrated that music therapy can allow a reduction in pain, fear-related stress [reduced salivary cortisol (113)], and anxiety outcomes (112). This can allow increased patient adherence toward medical procedures involved during cancer therapies and screening, for instance, screening mammography (114), sigmoidoscopy (115), colonoscopy (113), and even prostate biopsy (112, 116). Facilitation in the functioning of these mechanisms can have widespread influence on the regulation of cancer patient-related outcome and even the disease progression.

An additional outcome that can have important implications in management with auditory stimulations is the length of auditory-motor training duration. Here, interpretations can be drawn from neuroimaging research by Bangert and Altenmüller (117), and Ross et al. (106). Both the studies report that an auditory-motor training facilitates learning by acting on the rich neuroanatomical interconnectivity between the respective regions. The authors report a brief training duration lasting between 20 and 30 min to facilitate plasticity. Likewise, several of the published reviews and meta-analyses have also suggested a similar temporal course i.e., training session lasting for 25–40 min for auditory-motor training regimens (77, 78, 118). This training duration is relatively smaller as compared to conventional physiotherapy and physical exercise strategies discussed in the review by Duregon et al. (21). Therefore, beneficial implications in terms of cost-effectiveness and an enhanced prognosis in cancer survivors can be expected. Furthermore, we would also like to emphasize on the viability of the auditory stimulations, as a home-based intervention. Developing home-based interventions, are efficient for population groups in developing countries where lack of proper medical exposure accounts for widespread cancer-related morbidity and mortality (23). Wonders et al. (61) have also reported that home-based interventions can indeed impart beneficial effects in cancer survivors by reducing the peripheral neuropathic symptoms and enhancing the quality of life. We propose that in a home-based scenario patients can be taught by medical experts to utilize established smartphone rhythmic auditory cueing applications, such as Walkmate (119) to train gait effectively.

Finally, this perspective is a preliminary attempt to instigate scientific discussions for developing efficient rehabilitation protocols while using auditory neuroprosthetics based rehabilitation approach for enhancing motor recovery in patients with cancer. Incorporating these rehabilitation protocols with other sensory augmentation strategies such as virtual reality (120), joint prostheses (121–123), electrical stimulations (124) might have additional implications for enhancing the prognosis during cancer therapy. We have mentioned several mechanisms and findings from our previous review work, which could serve as the groundwork for future studies that could help design sensorimotor training regimens for the benefit of cancer population groups. Future studies are strongly recommended to analyze the effects of gait training with music-based auditory neuroprosthetics as a possible mechanism to counteract neurotoxic deficits because of cancer treatment.

## Author Contributions

SG conceptualized the perspective article. IG contributed in the formulation of the manuscript. Both authors approved the final draft.

### Conflict of Interest Statement

The authors declare that the research was conducted in the absence of any commercial or financial relationships that could be construed as a potential conflict of interest.
